# Socio-economic and demographic factors associated with fertility preferences among women of reproductive age in Ghana: evidence from the 2014 Demographic and Health Survey

**DOI:** 10.1186/s12978-020-01057-9

**Published:** 2021-01-02

**Authors:** Bright Opoku Ahinkorah, Abdul-Aziz Seidu, Ebenezer Kwesi Armah-Ansah, Edward Kwabena Ameyaw, Eugene Budu, Sanni Yaya

**Affiliations:** 1 School of Public Health, Faculty of Health, University of Technology Sydney, Sydney, Australia; 2 Department of Population and Health, University of Cape Coast, Cape Coast, Ghana; 3 College of Public Health, Medical and Veterinary Sciences, James Cook University, Townsville, QLD Australia; 4 School of International Development and Global Studies, University of Ottawa, Ottawa, Canada; 5 The George Institute for Global Health, The University of Oxford, Oxford, UK

**Keywords:** Fertility, Preferences, Ghana, Desire for children, Women, DHS

## Abstract

**Background:**

Understanding women’s desire to have more children is critical for planning towards future reproductive health behaviour. We examined the association between socio-economic and demographic factors and fertility preferences among women of reproductive age in Ghana.

**Methods:**

This  study used data from the 2014 Ghana Demographic and Health Survey. The sample consisted of 5389 women of reproductive age. We fitted Binary logistic regression models to assess the association between socio-economic status and fertility preferences, whiles controlling for demographic factors. The results were presented as crude odds ratios (cORs) and adjusted odds ratios (aORs) together with their corresponding 95% confidence intervals.

**Results:**

Approximately 60% of women of reproductive age in Ghana desired for more children. Women with no formal education were more likely to desire for more children compared to those with higher level of education (aOR = 2.16, 95% CI 1.29–3.48). The odds of desire for more children was higher among women who lived in rural areas compared to those who lived in urban areas (aOR = 1.24, 95% CI 1.01–1.53). With region, women who lived in the Northern region were more likely to desire for more children compared to those who lived in the Ashanti region (aOR = 4.03, 95% CI 2.69–6.04). Similarly, women who belonged to other ethnic groups were more likely to desire for more children compared to Akans (aOR = 1.78, 95% CI 1.35–2.35). The desire for more children was higher among women with 0–3 births compared to those with four or more births (aOR = 7.15, 95% CI 5.97–8.58). In terms of religion, Muslim women were more likely to desire for more children compared to Christians (aOR = 1.87, 95% CI 1.49–2.34).

**Conclusion:**

This study concludes that women in high-socio economic status are less likely to desire more children. On the other hand, women in the Northern, Upper East and those belonging to the Islamic religious sect tend to desire more children. To aid in fertility control programmes designing and strengthening of existing ones, these factors ought to be critically considered.

## Plain English summary

Understanding women’s desire to have more children is critical for planning towards future reproductive health behaviour. We examined the association between socio-economic and demographic factors and fertility preferences among women of reproductive age in Ghana.

This study used data from the 2014 Ghana Demographic and Health Survey. The women who participated in the study were 5389 and aged 15–49. We calculated the distribution of the socio-economic and demographic factors across fertility preferences using percentages. Next, we carried out a bivariate logistic regression analysis to examine the independent associations between the socio-economic and demographic variables and desire for more children. The results were presented using crude odds ratios. Finally, we fitted a multivariable logistic regression model to examine the socio-economic and demographic factors associated with  desire for more children. The results were presented as adjusted odds ratios. Statistical significance was determined using 95% confidence intervals. The results show that women belonging to the poorest wealth quintile and  those with no formal education were more likely to desire for more children compared to richest and women with higher education respectively. Desire for more children was less likely among women aged 45–49 compared to those aged 15–19. With region of residence, women who lived in the Northern region were more likely to desire for more children compared to those who lived in the Greater Accra region. Similarly, women who belonged to other ethnic groups were more likely to desire for more children compared to Akans. The desire for more children increased with lower parity as women with 0–3 births were more likely to desire for more children. This study concludes that women in high-socio economic status are less likely to desire more children. On the other hand, women in the Northern, Upper East and those belonging to the Islamic religious sect tend to desire more children. To aid in fertility control programmes designing and strengthening of existing ones, these factors ought to be critically considered.

## Background

The size of a family is of great importance not only for a country but for the welfare and health of the individual, family and community [1]. The current demographic changes have witnessed unprecedented reduction in the level of fertility in all regions of the world [2]. This is relevant in low- and middle-income countries where population growth has adverse effects on poverty situation and wellbeing of the nations [3]. Fertility is the major determinant of high population growth and on a larger scale, population growth could hinder a country’s economic performance as this may put pressure on the already limited infrastructure and government funds [4, 5].

Many households with smaller family sizes are more likely to benefit from demographic dividends and this suggests that uncontrolled population growth will have some dire consequences on the economic status as well as other devastating effects on maternal and child health [4, 6]. High fertility coupled with high population growth may lead to increase in maternal and paediatric diseases and or mortality [7]. Attempts to control fertility or child birth have over the years focused on meeting contraceptive needs of the population and improving public health education on the knowledge, awareness and proper use of contraceptives [8]. Contraceptive use has helped to prevent about 2.7 million infant and maternal mortality globally [9].

The desire to have more children is important for understanding future reproductive behaviour [10]. In Ghana, the decrease in fertility from 6.4 in 1988 to 4.2 in 2014 may be due to government policies initiated to manage population growth [10]. Such policies include the Contraceptive Social Marketing (CSM) project (1987–1990), the Ghana Family Planning and Health Programme (FPHP) (1990–1996), the Ghana Population and AIDS Project (GHANAPA) (1996–2000) and 1994 and 2004 Ghana Population Policies [8].

Fertility preference is pivotal in the fertility tendencies and this is also influenced by numerous interrelated variables such as age, marital status, income, educational level and parity [11–13]. Socio-economic status, education, maternal age, number of children ever born, infant mortality, and opinion of key people about the number of children to bear have been identified to have significant influence on fertility preferences [14–17]. The 2014 Ghana Demographic and Health Survey (GDHS) indicates that women with secondary or higher education (28%) are less likely to want more children as compared with their counterparts with no formal education or with primary education [10].

In Ghana, socio-economic status and fertility preference influence reproductive health and behaviour of both men and women in their reproductive ages significantly [18]. A phenomenon that has received minimal attention in Ghana is the association between socio-economic status and fertility preferences among women of reproductive age. While several studies have been carried out on childhood mortality, intra-household bargaining power and fertility preferences among women in Ghana [3, 19, 20], to the best of our knowledge, very little evidence exist on the association between socio-economic status and fertility preferences among women of reproductive age in Ghana. Hence, in this study, we sought to examine the association between socio-economic and fertility preferences among women of reproductive age in Ghana, while controlling for relevant demographic factors. Findings from such a nationally representative study will be vital to aid in strengthening existing measures to tackle high fertility and improvement in maternal and child health.

## Methodology

### Data source

Data for this study was obtained from the 2014 Ghana Demographic and Health Survey (GDHS) [10]. This is a cross sectional study implemented by the Ghana Statistical Service (GSS), Ghana Health Service (GHS), and the National Public Health Reference Laboratory (NPHRL). Whilst the Inner City Fund (ICF) provided technical assistance, about nine organisations financed the study including the United States Agency for International Development (USAID), the United Nations Development Programme (UNDP) and the Ghana AIDS Commission (GAC). The 2014 GDHS offers an updated estimate of fundamental demographic and health information covered by the early surveys since its inception in 1988. The survey targeted women aged 15–49 and men aged 15–59. The sampling procedure follows a two-stage cluster sampling approach. Selection of sample points or clusters was done in the first stage. This yielded 427 clusters from rural (211) and urban (216) locations. Systematic sampling of households occurred in the second stage through random sampling approach. Detailed description of the sampling procedure and other methodological issues are extensively espoused in the report [10]. Weighting factors were added to the data files to ensure that computed results will be proportional at the national level. This was needful as the sample was not self-weighting at the national level. The sample for our study consisted of 5389 women of reproductive age who were married/cohabiting or had previously married.

### Derivation of study variables

#### Outcome variable

The outcome variable for the study is fertility preferences. This was derived from a question answered by women aged 15–49 during the 2014 GDHS. The question was framed as “would you like to have a (another) child with your husband/partner, or would you prefer not to have any more children with him?” Responses to this question was categorised into: “want a (another) child,” “want no more,” “cannot get pregnant,” “undecided,” and “don't know.” Our outcome variable was computed from two of these responses namely “want a (another) child,” coded as 1 and “want no more,” coded as 0. Women who provided “undecided,” and “don't know” responses were excluded because their responses were unclear about their fertility preference and those who indicated “cannot get pregnant were not at risk of getting pregnant. These reasons necessitated the decision to drop them from our sample in other not to get biased estimates.

#### Explanatory variable

Socio-economic status is the cardinal explanatory variable for the study. Education and wealth status were used as proxies for socio-economic status. Whilst varied indicators are used for measuring socio-economic status, these two indicators (education and wealth) have dominated in the literature over time [21–23]. Education as used in the DHS, focuses on highest level of education that a person has completed. We used education of both the women and their partners. This is categorised into no education, primary, secondary and higher. Wealth quintile on the order hand, is measured as an ordinal variable ranging from poorest to richest. It is a composite measure that is computed by identification of household assets such as bicycle and television. Through Principal Component Analysis (PCA), these assets are transformed into an index and categorised into poorest, poorer, middle, richer and richest [24]. We also considered some critical socio-demographic characteristics of the sampled women. These are age, residence, occupation, region, ethnicity, parity, religion and access to mass media (newspaper/magazine, radio and television) (see Table 1).

**Table 1:** Fertility preferences across women’s socio-economic status and their demographic characteristics. Source: Computed from 2014 GDHS

Variables	Weighted N	Weighted %	Fertility preferences	
			Want no more (40.3%)	Want a (another) child (59.7%)
Socio-economic variables				
Wealth quintile				
Poorest	1001	18.6	35.3	64.7
Poorer	961	17.8	52.6	47.4
Middle	1080	20.0	45.6	54.4
Richer	1110	20.6	40.1	59.9
Richest	1238	23.0	37.1	62.9
Level of education				
No education	1429	26.5	42.6	57.4
Primary	980	18.2	48.0	52.0
Secondary	2697	50.1	39.7	60.3
Higher	283	5.3	37.0	63.0
Partner’s level of education				
No education	1094	20.3	38.4	60.6
Primary	473	8.8	41.7	58.3
Secondary	3181	59.0	44.3	55.7
Higher	640	11.9	33.8	66.2
Demographic variables				
Age				
15–19	105	2.0	5.8	94.2
20–24	621	11.5	9.1	90.9
25–29	1074	19.9	17.5	82.5
30–34	1105	20.5	34.3	65.7
35–39	1028	19.1	50.5	49.5
40–44	810	15.0	72.2	27.8
45–49	646	12.0	80.9	19.1
Residence				
Urban	2747	51.0	41.0	59.0
Rural	2642	49.0	42.8	57.2
Occupation				
Not working	663	12.3	32.7	67.3
Managerial	370	6.9	29.9	70.1
Clerical	48	0.9	38.3	61.7
Sales	2246	41.7	43.3	56.7
Agricultural	1320	24.5	48.6	51.4
Household	27	0.5	36.7	63.3
Manual	715	13.3	39.9	60.1
Region				
Western	536	9.9	45.2	54.8
Central	552	10.24	48.0	52.0
Greater Accra	1045	19.4	42.4	57.6
Volta	431	8.0	52.9	47.1
Eastern	527	9.8	50.7	49.3
Ashanti	972	18.0	43.7	56.3
Brong Ahafo	418	7.8	40.1	59.9
Northern	554	10.3	21.2	78.9
Upper East	223	4.1	33.0	67.0
Upper West	133	2.5	33.7	66.3
Ethnicity				
Akan	2591	48.1	47.4	52.7
Ga-Adangbe/Ewe	1142	21.2	51.0	49.0
Mole-Dagbani	849	15.8	31.3	68.7
Others	807	15.0	28.5	71.5
Parity				
0–3 births	3147	58.4	21.0	79.0
Four or more births	2242	41.6	71.1	28.9
Religion				
Christianity	4225	78.4	43.9	56.1
Islam	862	16.0	26.5	73.5
Traditionalist	141	2.6	41.0	59.0
No religion	161	3.0	42.6	57.4
Frequency of reading newspaper/magazine				
Not at all	4694	87.1	42.7	57.3
Less than a week	359	6.7	38.4	61.6
At least once a week	336	6.2	33.9	66.1
Frequency of listening to radio				
Not at all	837	15.5	43.0	57.0
Less than once a week	1744	32.4	43.1	56.9
At least once a week	2808	52.1	40.7	59.3
Frequency of watching television				
Not at all	1421	26.4	48.4	51.6
Less than once a week	1352	25.1	40.0	60.0
At least once a week	2616	49.6	39.2	60.8

### Data analyses

Data for this study were analysed using Stata 14.2 for Windows. First, we calculated the distribution of the socio-economic and demographic factors across fertility preferences using percentages (see Table 1). After this, we checked for multicollinearity among the explanatory variables, using the variance inflation factor (VIF) and the results showed no evidence of high collinearity (Mean VIF = 1.72, maximum VIF = 3.47, and minimum VIF = 1.10). Next, we carried out a bivariate logistic regression analysis to examine the independent associations between the socio-economic and demographic variables and desire for more children. The results were presented using crude odds ratios (cOR). Finally, we fitted a multivariable logistic regression model to examine the socio-economic and demographic predictors of desire for more children. The results were presented as adjusted odds ratio (aORs) together with their corresponding 95% confidence intervals signifying level of precision. On the account that the sample was not self-weighting, we applied sample weight in our computations whilst the survey (svy) command was used to cater for the study design.

### Ethical consideration

The survey was ethically approved by the ICF International and Ethical Review Committee of Ghana Health Service [10]. We did not need ethical permission for this study since the DHS datasets are publicly accessible. All ethical procedures were, however, observed during to survey to align the study with the regulations for the protection of human rights as required by the U.S. Department of Health and Human Services. More details regarding DHS data and ethical standards are available at: http://goo.gl/ny8T6X.

## Results

Table 1 presents results on fertility preferences across women’s socio-economic status and their demographic characteristics. Approximately 60% of women of reproductive age in Ghana desired for more children. We found that the desire for more children was high among poorest women (64.4%), women with higher level of education (63.0%), those whose partners had higher level of education (66.2%), women aged 15–19 (94.2%), urban residents (59.0%), women in managerial jobs (70.1%) and women in the Northern region (78.9%). Similar observation was made among women who belonged to other ethnic groups (71.5%), those with parity 0–3 (79.0%), Muslims (73.5%), those who read newspaper/magazine at least once a week (66.1%), those who listened to radio at least once a week (59.3%) and those who watched television at least once a week (60.8%).

Model 1 of Table 2 shows results of the unadjusted odds ratios of the socio-economic and demographic predictors of desire for more children. All the variables showed statistically significant associations with desire for more children at the crude level. The second model of Table 2 presents results of the multivariable logistic regression analysis of the socio-economic and demographic predictors of fertility preferences among women of reproductive age in Ghana, while controlling potential covariates. The results indicate that women with no formal education were more likely to desire for more children compared to those with higher level of education (aOR = 2.16, 95% CI 1.29–3.48). Desire for more children was less among women aged 45–49 compared to those aged 15–19 (aOR = 0.04, 95% CI 0.02–009). The odds of desire for more children was higher among women who lived in rural areas compared to those who lived in urban areas (aOR = 1.24, 95% CI 1.01–1.53). With region, women who lived in the Northern region were more likely to desire for more children compared to those who lived in the Ashanti region (aOR = 4.03, 95% CI 2.69–6.04). Similarly, women who belonged to other ethnic groups were more likely to desire for more children compared to Akans (aOR = 1.78, 95% CI 1.35–2.35). The desire for more children was higher among women with 0–3 births compared to those with four or more births (aOR = 7.15, 95% CI 5.97–8.58). In terms of religion, Muslim women were more likely to desire for more children compared to Christians (aOR = 1.87, 95% CI 1.49–2.34). Women who never watched television were less likely to desire for more children compared to those watched television at least once a week (aOR = 0.76, 95% CI 0.61–0.95).

**Table 2:** Socio-economic and demographic factors associated with fertility preferences among women of reproductive age in Ghana. Source: Computed from 2014 GDHS

Variables	Model 1 cOR 95% CI	Model 2 aOR 95% CI
Wealth quintile		
Poorest	1.06 [0.89–1.26]	1.02 [0.67–1.56]
Poorer	0.62*** [0.51–0.74]	0.85 [0.60–1.21]
Middle	0.73** [0.61–0.88]	0.82 [0.61–1.11]
Richer	0.97 [0.80–1.17]	0.91 [0.70–1.17]
Richest	Ref	Ref
Level of education		
No education	0.64** [0.48–0.86]	2.16** [1.29–3.48]
Primary	0.59** [0.44–0.80]	1.90** [1.17–3.09]
Secondary	0.72* [0.54–0.95]	1.80** [1.17–2.78]
Higher	Ref	Ref
Partner’s level of education		
No education	0.73** [0.60–0.90]	0.80 [0.57–1.13]
Primary	0.75* [0.59–0.96]	0.93 [0.64–1.35]
Secondary	0.61*** [0.51–0.74]	0.77 [0.59–1.02]
Higher	Ref	Ref
Age		
15–19	Ref	Ref
20–24	0.66 [0.29–1.49]	0.67 [0.30–1.53]
25–29	0.38* [0.17–0.83]	0.52 [0.23–1.16]
30–34	0.13*** [0.06–0.29]	0.30** [0.14–0.67]
35–39	0.07*** [0.03–0.14]	0.18*** [0.08–0.39]
40–44	0.03*** [0.01–0.05]	0.07*** [0.03–0.17]
45–49	0.01*** [0.01–0.03]	0.04*** [0.02–0.09]
Residence		
Urban	Ref	Ref
Rural	0.93* [0.84–0.99]	1.24* [1.01–1.53]
Occupation		
Not working	1.79*** [1.49–2.16]	0.91 [0.70–1.17]
Managerial	1.72*** [1.33–2.21]	1.43 [0.98–2.10]
Clerical	1.89 [0.93–3.82]	1.38 [0.64–2.10]
Sales	Ref	Ref
Agricultural	0.88 [0.77–1.01]	0.97 [0.77–1.22]
Household	1.24 [0.60–2.54]	1.37 [0.65–1.26]
Manual	1.20* [1.01–1.43]	1.01 [0.81–1.26]
Region		
Western	0.89 [0.70–1.14]	1.15 [0.82–1.60]
Central	0.80 [0.63–1.01]	1.10 [0.79–1.53]
Greater Accra	Ref	Ref
Volta	0.65** [0.51–0.84]	0.84 [0.58–1.22]
Eastern	0.72** [0.56–0.91]	1.03 [0.74–1.45]
Ashanti	0.95 [0.74–1.20]	1.57** [1.13–2.19]
Brong Ahafo	1.10 [0.86–1.40]	1.47* [1.04–2.08]
Northern	2.74*** [2.14–3.52]	4.03*** [2.69–6.04]
Upper East	1.49** [1.17–1.91]	1.63* [1.08–2.47]
Upper West	1.45** [1.11–1.88]	1.90** [1.24–2.92]
Ethnicity		
Akan	Ref	Ref
Ga-Adangbe/Ewe	0.86 [0.74–1.01]	1.04 [0.81–1.33]
Mole-Dagbani	1.97*** [1.71–2.28]	1.45* [1.07–1.97]
Others	2.25*** [1.92–2.64]	1.78*** [1.35–2.35]
Parity		
0–3 births	9.08*** [8.01–10.30]	7.15*** [5.97–8.58]
Four or more births	Ref	Ref
Religion		
Christianity	Ref	Ref
Islam	2.17*** [1.87–2.52]	1.87*** [1.49–2.34]
Traditionalist	1.12 [0.82–1.53]	1.40 [0.92–2.12]
No religion	1.05 [0.78–1.41]	1.55* [1.06–2.27]
Frequency of reading newspaper/magazine		
Not at all	0.68** [0.53–0.89	0.91 [0.65–1.27]
Less than a week	0.87 [0.62–1.23]	0.93 [0.62–1.40]
At least once a week	Ref	Ref
Frequency of listening to radio		
Not at all	0.95 [0.81–1.01]	0.95 [0.76–1.18]
Less than a week	0.86* [0.76–0.97]	0.91 [0.77–1.08]
At least once a week	Ref	Ref
Frequency of watching television		
Not at all	0.74*** [0.66–0.84]	0.76* [0.61–0.95]
Less than once a week	0.87 [0.75–1.00]	1.14 [0.93–1.39]
At least once a week	Ref	Ref
*N*	5389	5389
Pseudo *R*^2^		0.34

Exponentiated coefficients; 95% confidence intervals in brackets

*cOR* crude odds ratio, *aOR* adjusted odds ratio

*p < 0.05, **p < 0.01, ***p < 0.001

## Discussion

This study sought to assess the association between socio-economic and demographic factors and fertility preferences among women of reproductive age in Ghana. The results from the study showed that women in the richest wealth quintile and those with higher level of education are less likely to desire more children. These findings are in line with previous studies that higher socio-economic status is associated with lower fertility desires. Specifically, similar findings have been observed in Nigeria [25], India [26] and various multi-country studies [27–30]. However, our results deviate from what was found in a Ugandan study by Matovu et al. [31]. They found that, those in lower socio-economic status (education), thus having primary education were associated with higher fertility desire than those with no formal education. The possible reasons for the variation in study findings could be differences in cultural believes, levels of socio-economic development and the importance ascribed to children in the various settings. Nonetheless, Matovu et al. [31] suggested a qualitative study to unravel such nuances. This therefore implies that women with low level of education should be educated on the importance associated with low fertility.

The current findings on the association between socio-economic status and fertility preferences can be discussed within the context of the wealth flow hypotheses postulated by Caldwell [16, 17]. The theory explains that in modern societies those who are in high socio-economic strata tend to interpret more children as additional burden which can strain their resources including time as opposed to those in the low socio-economic status who wish to have more children with the notion that the children will serve as their old age security. In addition, due to the various economic activities of those in high socio-economic activities, they might not have more children as opposed to those who are in the low socio-economic status. Another plausible explanation by Channon and Harper [32] is that in contemporary era, women have competing life goals. They maintained that for highly educated women, it is sometimes problematic for them to combine many children and life goals such as occupying certain managerial position that will not allow certain amount of maternity leave within a given period.

Aside the socio-economic factors, it is worthwhile to discuss the association found among the demographic factors and desire for more children. The study showed that as parity increased, the less likely it is for women to indicate that they want more children. This is congruent with several previous studies in other parts of the world such as Nigeria [33, 34], China [35] and Sri Lanka [36]. The probable explanation to this finding is that some of these women might have achieved their desired number of children.

Our study also showed a statistically significant association between region of residence, ethnicity, religious affiliation and fertility desires. Specifically, those in the northern part of Ghana (Northern and Upper West), those who belonged to the Mole-Dagbani ethnic group and those who are Muslims tend to have higher odds of desiring more children. Several studies have been conducted on the socio-cultural beliefs and practices surrounding fertility behaviour in Ghana [37]. In Ghana, the northern part is predominantly occupied by the Mole Dagbani’s of whom majority are also Muslims. In terms of religion some scholars have explained that stronger religious beliefs tend to correlate positively with a stronger desire more children [38, 39]. A recent qualitative study conducted by Abdi et al. [40] in two Muslim communities in Kenya found that Muslim women tend to desire more children due to the belief that children are a blessing from God. Due to this some of them would wish to receive more of the blessings God has in stock for them in the form of children. Another possible explanation is that some Muslim women who are in polygamous marriages tend to compete in terms of the number of children to ensure the continuity of their husband’s lineage and to enhance their own bargaining power and ‘value’ in the relationship [41]. The patriarchal nature of the northern societies and the various ethnic groups places so much credence on large family sizes [37] which might influence the women’s’ views on desire for more children. This therefore suggest the need for more education targeting various community leaders, family heads and husbands to appreciate the importance of smaller family sizes by adopting various contraceptives. These can be achieved through education during community durbars and other social gatherings.

## Strengths and limitations

This study derives its strength from the use of nationally representative dataset that employed multi-stage sampling technique to select the respondents. With this, the findings are generalisable to all women in their reproductive age in Ghana to a greater extent. The relatively large sample size also aided in fitting robust logistic regression models to model the association between socio-economic status and desire for more children while controlling for confounders. Despite these strengths, the study’s results must be interpreted cautiously against the following limitations. First, it is impossible to establish temporality of sequence. Second, the possibility of social desirability biases cannot be overruled.

## Conclusion

This study concludes that women in high-socio economic status are less likely to desire for more children. On the other hand, women in the Northern, Upper East and those belonging to the Islamic religious sect tend to desire more children. To aid in fertility control programmes deigning and strengthening existing ones, these factors ought to be critically considered. It is crucial to educate those with low level of education on the importance of attaining fewer children by adopting various family planning strategies including contraceptive usage.

## Data Availability

Data for this study were sourced from Demographic and Health surveys (DHS) and available here: http://dhsprogram.com/data/available-datasets.cfm.

## Abbreviations

ANC: Antenatal care
DHS: Demographic and Health Surveys
cOR: Crude odds ratios
aOR: Adjusted odds ratio
CI: Confidence interval
GHS: Ghana Health Service
GDHS: Ghana Demographic and Health Survey
WHO: World Health Organization
ICF: Inner City Fund
USAID: United States Agency for International Development
UNDP: United Nations Development Programme
GAC: Ghana AIDS Commission
PCA: Principal Component Analysis
